# Auricular Acupuncture for Craving in a Single-subject Case Study of Woman with Fibromyalgia and Chronic Ecstasy Use

**Published:** 2018-07

**Authors:** Bijan PIRNIA, Kambiz PIRNIA, Alireza ZAHIRODDIN

**Affiliations:** 1. Dept. of Psychology, Faculty of Humanities, University of Science and Culture, Tehran, Iran; 2. Behavioral Sciences Research Center, Shahid Beheshti University of Medical Sciences, Tehran, Iran; 3. Bijan Center for Substance Abuse Treatment, Tehran, Iran; 4. Dept. of Psychiatry, Behavior Research Center, Shahid Beheshti University of Medical Sciences, Tehran, Iran

## Dear Editor-in-Chief

**Fig. 1:**
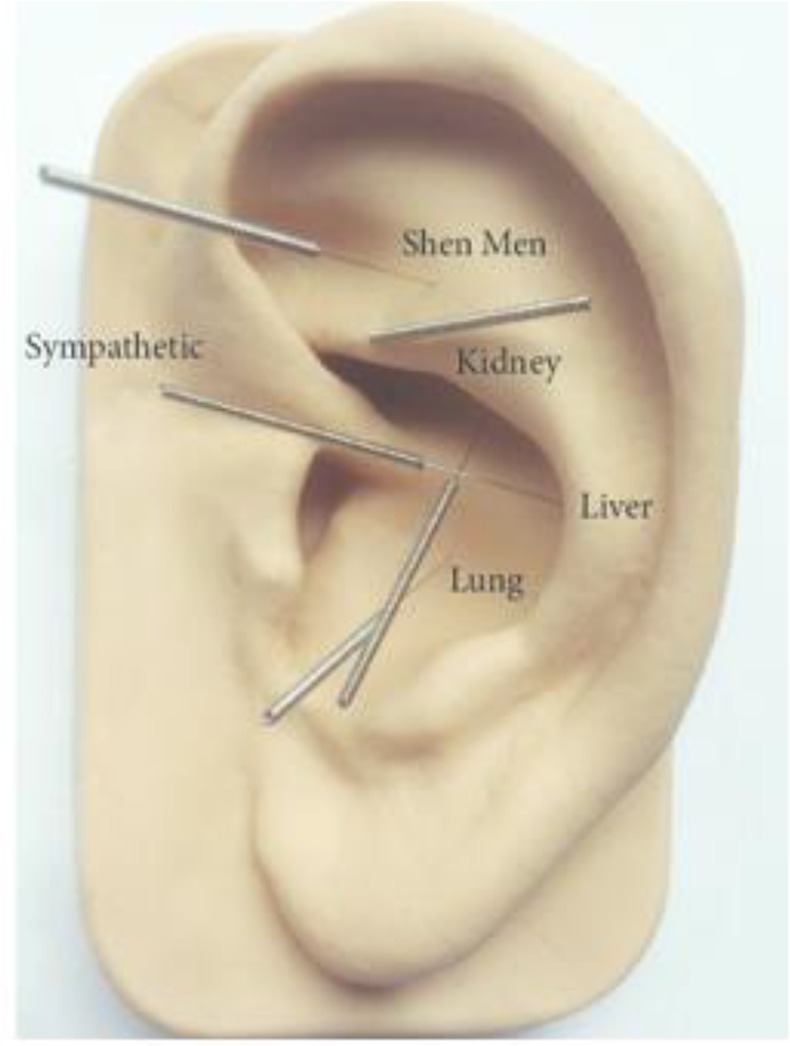
Acupuncture points according to the NADA protocol

Acupuncture is a treatment method used for two thousand years (1). Auricular acupuncture (AA) is a specific type of acupuncture first described in 2500 BC and is presently executed in National Association Protocol of Acupuncture (NADA, Fig. 1) in 250 hospitals in Great Britain and the United States. NADA Protocol is an evidence-based approach and client-centered (2).

Fibromyalgia syndrome (MJ 42.12, ICD-11 Revision) is a chronic disease characterized by widespread pain, fatigue, and psychological problems. Ecstasy is a bunch of stimulants derived from Amphetamine that its dependency is associated with neurotoxicity.

This study aimed to assess the effectiveness of auricular acupuncture on reducing pain and craving in an ecstasy-dependent individual with fibromyalgia. This study was a single subject research using multiple baseline designs in the form of Pattern A-B (TCTR20180607002). Data for this study were collected from Mar to Nov 2016.

The patient was a 38-yr-old woman who was a chronic ecstasy user and had been taking ecstasy tablets for three years continuously. She was referred to Bijan Clinic, Tehran, Iran with a diagnosis of fibromyalgia and experienced musculoskeletal pain and with the aim of use craving. The acupuncture was performed for three weeks and in the form of three weekly sessions, and the studied indices were evaluated as repeated measurements. During the treatment process, five points of ear according to the NADA protocol were subjected to manual stimulation with stainless steel disposable needles (0.25+13 mm) with a depth of 2–3 mm.

The entire process was carried out based on the latest version of the Declaration of Helsinki and informed consent was taken from the patient.

In this study, structured clinical interviews, demographic researcher-made questionnaires, Usage Craving Questionnaire and Visual Analogue Scale were used.

**Fig. 2:**
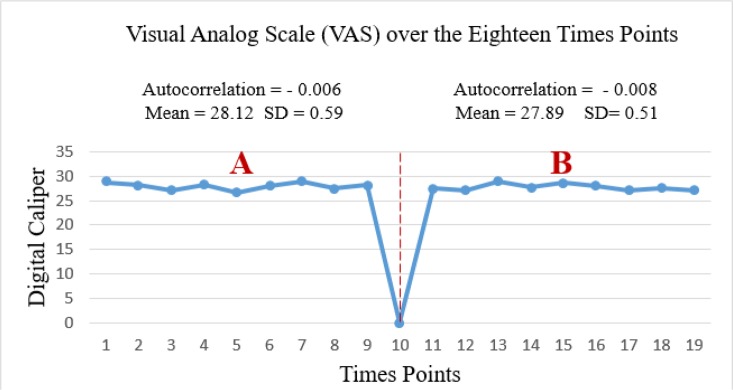
Visual analog scale over the eighteen-time points

**Fig. 3:**
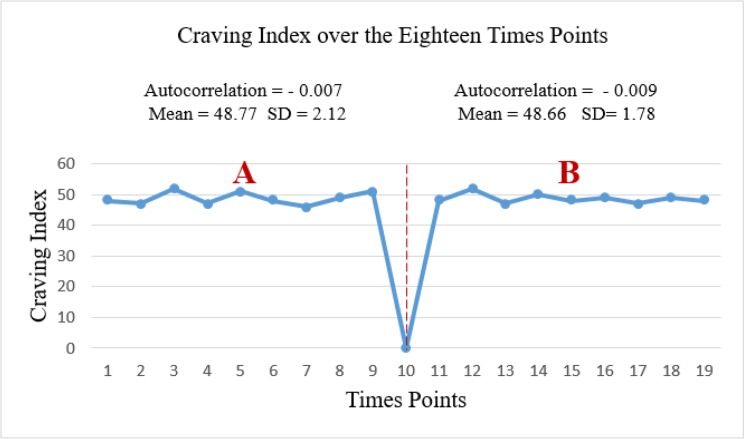
Craving Index over the eighteen-time points

The primary outcome showed that AA had not a significant effect on reducing the pain (A mean of 28.12 ± 0.59 in left vs*.* a mean of 27.89 ± 0.51 in right, *P*>0.05; Fig. 2). Moreover, secondary outcome showed that there was not a significant reduction in craving index (A mean of 48.77 ± 2.12 in left vs. a mean of 48.66 ± 1.87 in right, *P*>0.05; Fig. 3).

Our results showed that acupuncture had not any significant effect on reducing pain. Consistent with these results, a meta-analysis showed that acupuncture did not have a significant effect on reducing musculoskeletal pain (3).

In a narrative review (4), the amount and quality of published researches on the effectiveness of acupuncture in reducing chronic pain resulting from dependence to opioids reported inadequate and the use of this method in the field of evidence-based treatment reported inexcusable.

Consistent with our results, acupuncture did not have a significant effect on reducing craving (5). No evidence on the effectiveness of acupuncture in reducing drug use in drug abusers with psychiatric disorders was observed at the same time (6).

In explaining the findings of the present study, it is needed hormonal changes due to stimulant usage be attended. Chronic use of stimulus affects the production of TH. In order to investigate the effectiveness of acupuncture on levels of TH, acupuncture had not a significant effect on reducing death of tyrosine hydroxylase cells in the animal example (7). Increasing in the death of TH cells reduces the secretion of dopamine and limits the analgesic effects of these hormones and this process can be challenging for the effectiveness of the treatment. This failure in interaction with undesirable growth components, such as early onset of stimulants use, can lead the way for the formation of psychological damages. The evaluation of the effectiveness of acupuncture as a complementary therapy in ecstasy dependents requires more trials.
